# Subaxial Cervical Sagittal Alignment as an Anatomical Correlate of Obstructive Sleep Apnea Severity: A Cephalometric Analysis

**DOI:** 10.3390/biomedicines14091998

**Published:** 2026-09-05

**Authors:** Ali Osman Korkmaz, Kader Aydın, Alper Atasever

**Affiliations:** 1 Clinical Anatomy Program, Graduate School of Health Sciences, Istanbul Medipol University, Istanbul 34810, Türkiye; 2 Department of Oral and Maxillofacial Radiology, Istanbul Medipol University, Istanbul 34810, Türkiye; 3 Department of Anatomy, Istanbul Medipol University, Istanbul 34810, Türkiye

**Keywords:** Obstructive sleep apnea syndrome, cervical lordosis, cephalometry, Apnea–Hypopnea Index, posture

## Abstract

**Background:** Obstructive sleep apnea syndrome (OSA) is a significant disorder characterized by recurrent upper airway obstruction. Cervical vertebral alignment and lordosis angles may influence airway cross-sectional area. This study aimed to investigate the relationship between cervical lordosis angles and disease severity in patients with OSA. **Methods:** The study included 30 patients with OSA (mild *n* = 6, moderate *n* = 11, severe *n* = 13 based on Apnea–Hypopnea Index [AHI]) and 30 non-OSA controls presenting with mechanical neck pain. C1–C2, C1–C7, and C2–C7 angles were measured using the Cobb method on lateral cervical cephalometric radiographs. Additional soft tissue and airway parameters — prevertebral soft-tissue thickness at C2 (PST-C2) and C4 (PST-C4), posterior airway space (PAS), and mandibular plane–hyoid distance (MP-H)— were measured as anatomical surrogates given the unavailability of BMI data. Multiple linear regression (ANCOVA) models adjusting for age and sex were constructed for each cervical angle. **Results:** All cervical angles were significantly reduced in patients with OSA compared to controls (C1–C2: 25.51 ± 6.47° vs 33.3 ± 4.44°; C1–C7: 35.0 ± 11.9° vs 51.2 ± 7.34°; C2–C7: 9.19 ± 10.6° vs 19.9 ± 5.05°; all *p* < 0.001). Within the OSA group, a significant moderate negative correlation was found between AHI and the C2–C7 angle (ρ = − 0.522, *p* = 0.003), while the C1–C7 angle showed a borderline association (ρ = −0.361, *p* = 0.050) and C1–C2 did not reach significance (ρ = +0.118, *p* = 0.534). After adjustment for age and sex, group membership (OSA vs. control) remained a strong, independent predictor of all three cervical angles (all *p* < 0.001), whereas neither age nor sex was a significant predictor (all *p* > 0.34). PST-C2 was significantly higher in the OSA group (4.93 ± 1.06 mm vs. 4.30 ± 0.70 mm, *p* = 0.017), whereas PST-C4, PAS, and MP-H did not differ significantly between groups (all *p* > 0.05), and none of these parameters correlated significantly with the C2–C7 angle. **Conclusions:** Significant cervical lordosis loss was observed in patients with OSA compared to non-OSA controls, with the C2–C7 angle showing a significant negative correlation with AHI within the OSA group; this association remained robust after adjustment for age and sex. Supplementary soft tissue and airway measurements showed no significant correlation with cervical alignment or AHI, suggesting the observed association is not simply attributable to regional soft-tissue thickness or airway narrowing; however, without direct BMI data, a confounding role of systemic adiposity cannot be excluded. These findings suggest a potential association between cervical posture and upper airway dynamics; however, larger prospective studies are required to confirm clinical utility.

## 1. Introduction

Obstructive sleep apnea syndrome (OSA) is a common respiratory disorder characterized by recurrent upper airway collapse during sleep [1,2], leading to episodes of apnea or hypopnea accompanied by hypoxia. OSA affects 9–38% of the adult population and can lead to serious complications, including cardiovascular disease, metabolic disorders, and neurocognitive impairment [3,4,5].

The pathophysiology of OSA is multifactorial. Anatomical factors such as a narrow pharynx, macroglossia, retrognathia or micrognathia, low-positioned hyoid bone, and hypertrophic tonsils contribute to upper airway narrowing [6,7,8,9]. Additionally, non-anatomical factors—including decreased muscle tone during sleep, weakened muscle response to stimuli, hypersensitivity of the respiratory control system (high loop gain), and low arousal threshold—contribute to airway collapse and obstruction during sleep [3,10,11].

The cervical vertebrae function as fundamental supporting structures that determine neck position. The muscles in this region are related to the vertebrae either directly or through fascial structures [12,13]. Changes in vertebral alignment and angles may affect respiratory tract cross-sectional area and volumes [14,15,16].

Previous studies have examined the relationship between head position, upper cervical vertebra position, and airway cross-sectional area and volumes [13,17,18,19]. Cervical vertebra biomechanics demonstrate that the upper cervical vertebrae (C1–C2) primarily perform rotational movements, while the lower cervical vertebrae can also perform movements in the sagittal plane [13,20].

Notably, studies on OSA have determined that the segments where obstruction occurs correspond more frequently to the levels of the lower cervical vertebrae [6,21,22]. Cervical vertebral movement in the sagittal plane may affect pharyngeal muscle tone and volume, thereby altering upper airway patency [14,15,16,23,24]. In light of these anatomical and functional relationships, we designed this study to examine the relationship between Apnea–Hypopnea Index (AHI) values and cervical lordosis angular changes in patients with OSA, using a symptomatic non-OSA control cohort to discern whether OSA-related airway dynamics possess a distinct impact on cervical alignment beyond localized biomechanical factors.

## 2. Materials and Methods

### 2.1. Ethical Approval

This study was approved by the Istanbul Medipol University Non-Interventional Ethics Committee on 8 March 2017 (decision number: 91). Informed consent was obtained from all participants.

### 2.2. Study Population

Between June 2017 and July 2018, 30 patients diagnosed with OSA following examination and diagnostic procedures at the Polysomnography Laboratory of the Department of Chest Diseases at Istanbul Medipol University Bağcılar Mega Hospital were enrolled. Diagnosis was established by polysomnography, and patients were stratified into three groups according to AHI values: mild (AHI 5–15, *n* = 6), moderate (AHI 15–30, *n* = 11), and severe (AHI > 30, *n* = 13). Hypopnea was defined as ≥30% reduction in airflow accompanied by ≥3% oxygen desaturation or arousal, per AASM (American Academy of Sleep Medicine) 2012 criteria [25,26].

Patients with cancer, neuromuscular disease, prior head and neck surgery, or pregnancy were excluded. Additionally, individuals with cervical degenerative disease (spondylosis, osteoarthritis), inflammatory arthropathy (rheumatoid arthritis, ankylosing spondylitis), history of cervical trauma or fracture, or any systemic musculoskeletal disorder that could independently affect cervical alignment were excluded from both groups. As a control group, 30 individuals who presented to the same hospital with non-specific neck pain complaints between 2023 and 2024 were included. Control subjects were selected following radiological evaluation by a radiologist, and only those in whom no pathology affecting cervical lordosis or posture was identified were deemed eligible. The same exclusion criteria applied to the OSA group were also applied to the control group. All radiographs in both groups were obtained using the same standardized cephalometric equipment and imaging protocol throughout both recruitment periods. Because the two recruitment periods were separated by several years, we additionally verified, for each of the 60 subjects individually, that linear measurements were not affected by any magnification or scaling inconsistency: a known reference distance on each radiograph was measured in 3D Slicer (version 5.12.01), and the resulting pixel-to-millimeter scaling factor was applied by directly editing the voxel spacing values in the Volume Information module, so that all linear parameters reflect true real-world dimensions independent of acquisition period or workstation settings. The three cervical Cobb angles (C1–C2, C1–C7, C2–C7) are angular measurements derived from line-to-line relationships and are therefore inherently unaffected by any such calibration or magnification differences.

### 2.3. Radiographic Evaluation

Lateral cervical cephalometric radiographs were obtained using a digital extraoral cephalometric X-ray unit (Planmeca ProMax 2D; Planmeca Oy, Helsinki, Finland) according to a standard protocol [17,18,27]. Images were acquired with patients standing upright, shoulders in a relaxed position, arms at the sides, and the Frankfurt plane parallel to the ground [27]. Head position was standardized by placing a spirit level at the Frankfurt plane during imaging.

In addition to cervical lordosis angles, four soft tissue and airway parameters were measured on the same lateral cephalometric radiographs: prevertebral soft-tissue thickness at the C2 level (PST-C2), prevertebral soft-tissue thickness at the C4 level (PST-C4), posterior airway space (PAS), and the mandibular plane–hyoid distance (MP-H). PST-C2 and PST-C4 were measured as the shortest distance between the posterior pharyngeal wall and the anteroinferior border of the C2 and C4 vertebral bodies, respectively. PAS was measured as the shortest distance between the posterior pharyngeal wall and the base of the tongue along a line parallel to the Frankfurt horizontal plane. MP-H was defined as the perpendicular distance from the hyoid bone to the mandibular plane. All measurements were performed using ImageJ software (v1.52; National Institutes of Health, Bethesda, MD, USA) on the same standardized lateral cephalometric radiographs used for cervical angle assessment. Two additional sagittal alignment parameters were measured to provide a more comprehensive assessment of cervical sagittal balance: C7 slope, defined as the angle between the superior endplate of C7 and the horizontal reference line, and C2–C7 sagittal vertical axis (SVA), defined as the horizontal distance between a plumb line dropped from the centroid of C2 and the posterosuperior corner of C7. Both parameters were measured on the same lateral cephalometric radiographs used for cervical angle assessment.

### 2.4. Cervical Lordosis Measurement

**Figure 1:**
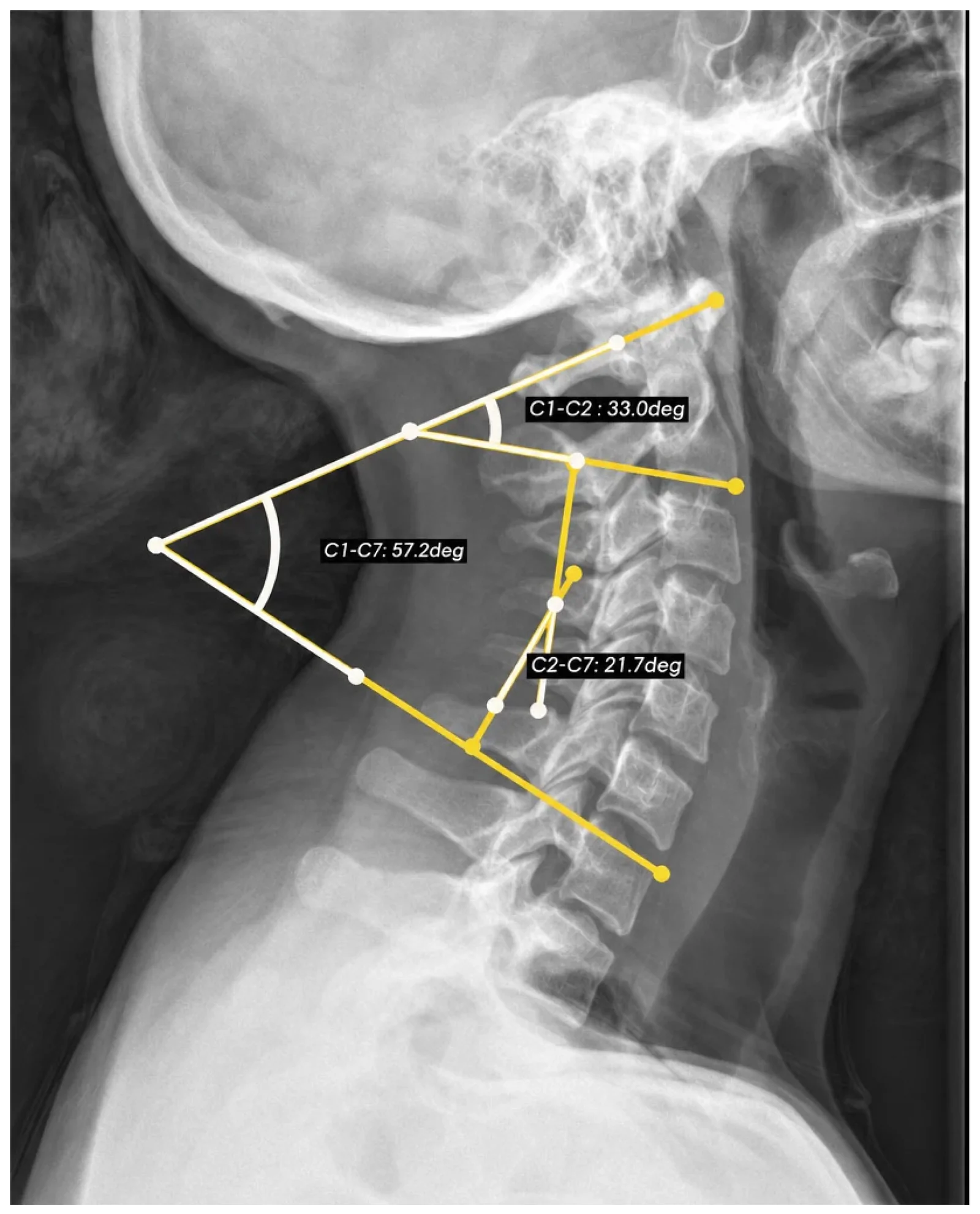
Lateral cervical cephalometric radiograph illustrating the angular measurements used to assess subaxial cervical sagittal alignment. The C1–C2 angle (33.0°) was measured between the line connecting the anterior and posterior tubercles of the atlas and the lower endplate line of C2. The C1–C7 angle (57.2°) was measured between the atlas tubercle line and the lower endplate line of C7. The C2–C7 angle (21.7°), calculated using the Cobb method, was measured between the lower endplate line of C2 and the upper endplate line of C7. All angles are marked with yellow reference lines on the radiograph.

C1–C2, C1–C7, and C2–C7 cervical lordosis angles were measured on lateral cephalometric radiographs. Measurements used the line connecting the midpoints of the anterior and posterior tubercles of the atlas (C1) as the superior reference line, the lower endplate line of C2 as the inferior reference line for the C1–C2 angle, the upper endplate line of C7 as the inferior reference line for the C1–C7 angle, and the lower endplate line of C2 together with the upper endplate line of C7 for the C2–C7 angle (Figure 1) [14,20]. This atlas tubercle-line method for the C1–C2 angle follows an approach previously described in the cephalometric literature [14,20]; however, it is less universally standardized than the C2–C7 Cobb method, which is more directly comparable across studies using endplate-to-endplate reference lines. (Figure 1) [14,20].

The Cobb measurement method was employed [20]. This method, defined by orthopedist John Robert Cobb, was originally developed to measure scoliosis curvatures on the frontal plane in anteroposterior radiographs and is now widely used for measuring sagittal plane curvatures. Measurements were performed using ImageJ software and PACS (Carestream Vue PACS v12.2.1.x) (Picture Archiving and Communication System) software.

### 2.5. Statistical Analysis

All statistical data computations and analyses were conducted using the jamovi statistical software (Version 2.4). Data normality was assessed using the Shapiro–Wilk test. Continuous variables are presented as mean ± standard deviation and median [IQR]( interquartile range). Between-group comparisons were performed using the Mann–Whitney U test and Kruskal–Wallis test. Correlation analyses were conducted using the Spearman’s correlation test. Correlation coefficients (ρ) were classified as very weak (0.00–0.20), weak (0.20–0.40), moderate (0.40–0.60), strong (0.60–0.80), and very strong (0.80–1.00). Fisher’s exact test was applied for categorical variables. Statistical significance was set at *p* < 0.05. To assess measurement reliability, intraclass correlation coefficients [ICC(2,1)] were calculated for all three cervical angle measurements in a randomly selected subsample of 15 participants per group. A two-way random effects model with absolute agreement was applied for single measures. ICC values were interpreted as poor (<0.50), moderate (0.50–0.75), good (0.75–0.90), or excellent (>0.90).

To evaluate whether between-group differences in cervical lordosis angles were independent of potential confounding by age and sex, multiple linear regression (ANCOVA) models were constructed for each cervical angle (C1–C2, C1–C7, C2–C7), with group (OSA vs. control), age, and sex included as independent variables (*n* = 60). Between-group comparisons for C7 slope and C2–C7 SVA were performed using the Mann–Whitney U test, given the non-normal distribution of the data, and effect sizes were reported using Cliff’s delta (δ). Associations between these two parameters and AHI were assessed using Spearman’s rank correlation coefficient (ρ), consistent with the statistical approach used for the other cervical alignment parameters. Body mass index (BMI) was not available for this retrospective cohort and could therefore not be included as a covariate.

## 3. Results

The demographic characteristics and cervical lordosis angle measurements are presented in Table 1. No significant difference was found in age between patients with OSA and controls (46.0 ± 11.3 years, median 44.0 [IQR 16.3] vs. 40.7 ± 15.6 years, median 37.5 [IQR 13.8]; *p* = 0.143). A statistically significant difference was observed in sex distribution (*p* = 0.001), with male predominance in the OSA group (26/30, 86.7%) compared with controls (13/30, 43.3%).

### 3.1. Cervical Lordosis Angles: OSA Group Versus Control Group

Statistically significant differences were observed in all three cervical lordosis angle measurements between patients with OSA and controls (all *p* < 0.001). The OSA group demonstrated markedly reduced cervical lordosis compared with controls across all segments. The C1–C2 angle was significantly lower in the OSA group (25.51 ± 6.47°, median 25.6°) compared with controls (33.3 ± 4.44°, median 33.0°; difference estimate: −7.44°, 95% CI: −10.64 to −4.26; *p* < 0.001; Cliff’s δ = −0.66). The C1–C7 angle was similarly reduced in the OSA group (35.0 ± 11.9°, median 34.0°) relative to controls (51.2 ± 7.34°, median 52.0°; difference estimate: −16.27°, 95% CI: −21.15 to −11.62; *p* < 0.001; Cliff’s δ = −0.80). The C2–C7 angle was also significantly lower in the OSA group (9.19 ± 10.6°, median 8.56°) than in controls (19.9 ± 5.05°, median 19.5°; difference estimate: −10.80°, 95% CI: −14.45 to −6.98; *p* < 0.001; Cliff’s δ = −0.69).

### 3.2. Cervical Lordosis Angles According to OSA Severity

The cervical lordosis angle measurements stratified by OSA severity subgroup are presented in Table 2. No significant difference was observed in the C1–C2 angle across OSA severity subgroups (mild: 23.5 ± 7.28°, median 25.9 [IQR 10.3]; moderate: 26.3 ± 5.31°, median 26.4 [IQR 8.76]; severe: 25.8 ± 7.28°, median 25.0 [IQR 11.3]; *p* = 0.739; ε^2^ = 0.02). Numerically lower C1–C7 angles were observed in the moderate and severe OSA subgroups compared with the mild subgroup; however, the overall Kruskal–Wallis test did not reach statistical significance (mild: 41.3 ± 10.8°, median 42.4; moderate: 34.5 ± 8.41°, median 36.5; severe: 32.5 ± 14.5°, median 29.7; *p* = 0.133; ε^2^ = 0.14). A statistically significant difference was found in the C2–C7 angle across severity subgroups (mild: 17.2 ± 4.85°, median 19.2; moderate: 8.02 ± 8.17°, median 10.4; severe: 6.49 ± 12.8°, median 5.17; *p* = 0.019; ε^2^ = 0.28). Post hoc pairwise comparisons using the Dwass–Steel–Critchlow–Fligner test revealed significant differences between the mild and moderate OSA subgroups and between the mild and severe OSA subgroups for the C2–C7 angle (both *p* < 0.05), whereas no significant difference was detected between the moderate and severe subgroups.

### 3.3. Measurement Reliability

Intraclass correlation coefficients demonstrated excellent inter-rater reliability for all three cervical angle measurements in both groups (Table 3). In the OSA group, ICC(2,1) values were 0.972 (95% CI: 0.921–0.991), 0.932 (95% CI: 0.811–0.977), and 0.922 (95% CI: 0.772–0.971) for the C1–C2, C1–C7, and C2–C7 angles, respectively. In the control group, ICC(2,1) values were 0.934 (95% CI: 0.831–0.979), 0.979 (95% CI: 0.935–0.992), and 0.968 (95% CI: 0.963–0.996) for the same angles. These results confirm the reproducibility of the cephalometric measurement protocol employed in this study.

### 3.4. Correlation Analyses

To examine the dose–response relationship between OSA severity and cervical alignment, Spearman’s rank correlation analysis was conducted within the OSA group only (*n* = 30) using continuous AHI values (Table 4). The C2–C7 angle demonstrated a significant moderate negative correlation with AHI (ρ = −0.522, *p* = 0.003), indicating that greater disease severity was associated with more pronounced subaxial lordosis reduction. The C1–C7 angle showed a borderline association (ρ = −0.361, *p* = 0.050), while the C1–C2 angle did not reach statistical significance (ρ = +0.118, *p* = 0.534). Correlation coefficients were classified according to the following thresholds: very weak (0.00–0.20), weak (0.20–0.40), moderate (0.40–0.60), strong (0.60–0.80), and very strong (0.80–1.00).

As a sensitivity analysis restricted to male participants (OSA: *n* = 26, controls: *n* = 13), all between-group differences remained statistically significant (all *p* ≤ 0.006), suggesting that the observed cervical lordosis reductions are unlikely to be primarily attributable to sex-related confounding. (Table 5)

### 3.5. Corresponding Results Text

Comparison of soft tissue and airway parameters between the OSA and control groups is summarized in Table 6. Prevertebral soft-tissue thickness at the C2 level (PST-C2) was significantly greater in the OSA group than in controls (4.93 ± 1.06 mm vs. 4.30 ± 0.70 mm, *p* = 0.017, Cliff’s δ = 0.363). No significant between-group differences were found for PST-C4 (*p* = 0.321), PAS (*p* = 0.154), or MP-H (*p* = 0.832).

Within the OSA group, none of the soft tissue or airway parameters showed a statistically significant correlation with AHI or with the C2–C7 cervical angle (Table 7; all *p* > 0.05). As these parameters are only indirect anatomical surrogates; this finding does not establish that adiposity or airway space do not confound the observed cervical alignment–OSA relationship; rather, it indicates that regional soft-tissue thickness and airway caliber at these specific cephalometric points are not themselves correlated with cervical angle or AHI within this cohort.

To address potential confounding effects of age and sex, multiple linear regression models were constructed for each cervical angle with group, age, and sex as covariates (Table 8; *n* = 60). Group membership remained a strong, statistically significant independent predictor of all three cervical angles after adjustment. Neither age nor sex independently predicted cervical angle values in any adjusted model (all *p* > 0.34).

The comparison of C7 slope and C2–C7 sagittal vertical axis (SVA) values between the OSA and control groups is presented in Table 9. The C7 slope was significantly lower in the OSA group compared with controls (24.14 ± 8.79°, median 20.9° vs. 27.68 ± 4.12°, median 26.95°; *p* = 0.015; Cliff’s δ = −0.37). Conversely, the C2–C7 SVA was significantly higher in the OSA group compared with controls (28.50 ± 9.54 mm, median 26.86 mm vs. 22.26 ± 7.95 mm, median 22.05 mm; *p* = 0.016; Cliff’s δ = +0.36). These findings indicate that both angular (reduced C7 slope) and translational (increased SVA) sagittal malalignment of the subaxial cervical spine co-occur in patients with OSA.

## 4. Discussion

This study demonstrated that cervical lordosis angles were significantly reduced in patients with OSA compared to controls, and that this reduction, particularly at the C2–C7 segment, showed a significant negative correlation with disease severity (ρ = −0.522, *p* = 0.003). Our findings indicate a potential association between reduced subaxial cervical lordosis and OSA, warranting further investigation into the role of cervical posture in upper airway dynamics.

Previous studies have examined the relationship between cervical posture and the upper airway. The literature has shown that cervical spine abnormalities, postural disorders, and neck pain are associated with OSA [15]. Similarly, Clavel et al. found cervical hyperextension and impaired posturo-respiratory coupling in OSA patients [16], emphasizing the effects of cervical vertebral postural changes on respiration.

Cephalometric analyses are widely used in OSA research [18,19,27]. Significant correlations have been found between cephalometric parameters and OSA severity [17,18]. Cervical–craniofacial skeletal morphology has been examined in detail through cephalometric evaluation in the literature [18]. However, evaluation of the lower cervical vertebrae has been insufficient in previous studies; our study aimed to contribute to the literature by focusing specifically on lower cervical vertebra angles.

Within-group correlation analysis revealed that the C2–C7 angle showed the strongest and only statistically significant correlation with OSA severity (ρ = −0.522, *p* = 0.003), while the C1–C7 angle showed a borderline association (ρ = −0.361, *p* = 0.050). These findings suggest that subaxial cervical alignment, particularly at the C2–C7 segment, has a more pronounced relationship with upper airway mechanics than the upper cervical region. Biomechanically, the lower cervical vertebrae (C2–C7) are capable of greater sagittal-plane motion than the upper cervical vertebrae (C1–C2) [13,20]; this feature may explain why lordotic changes are more pronounced in this region.

The C2–C7 angle also showed a statistically significant difference across OSA severity subgroups (*p* = 0.019; ε^2^ = 0.28), with values decreasing progressively from mild to severe. Post hoc analysis confirmed significant differences between the mild group and both the moderate and severe groups, but no significant difference between the moderate and severe groups. Notably, this pattern does not reflect a linear, progressive dose–response relationship: post hoc comparisons showed that the difference was driven entirely by the mild subgroup, with no further decline between the moderate and severe subgroups. This is more consistent with a threshold or floor effect—whereby cervical lordosis loss reaches a plateau once a certain severity level is exceeded—rather than a continuous, severity-proportional adaptation. This interpretation should also be considered in light of the small and unequal subgroup sizes (mild, *n* = 6; moderate, *n* = 11; severe, *n* = 13) and the markedly wider variability in the severe subgroup (SD = 12.8°, more than twice that of the moderate subgroup), which limits confidence in the shape of this relationship. With this caveat in mind, one possible explanation for such a threshold pattern is a segmental neuromuscular postural adaptation rather than a purely passive structural change. Several possible pathophysiological mechanisms may underlie this adaptation. First, proprioceptive loss in the cervical region has been reported in individuals with OSA [28], which may be a key factor triggering disturbances in head–neck posture. Second, hypertonicity of the accessory respiratory muscles, which are actively engaged in respiration, may directly affect this postural mechanism; increased respiratory effort in OSA patients raises the chronic tone of both the primary and accessory respiratory muscles. In particular, the anatomical location of the scalene muscle group shows that its origin extends down to the C3–C6 vertebral levels and inserts onto the 1st–2nd ribs [29]. Chronic hypertonicity of these muscles may limit thoracic movement to some degree, contributing to a reduction in cervical lordosis through a compensatory anatomical positional change [30]. This hypothesis and the associated levels of muscle contraction need to be validated in future studies with larger sample sizes using objective measurement methods such as electromyography. It is also important to emphasize that the cross-sectional design of this study precludes any determination of the direction of this association. While we have discussed potential mechanisms by which OSA could lead to cervical postural change, the reverse pathway is equally plausible: chronic intermittent hypoxia, altered respiratory effort, and sleep fragmentation associated with OSA could independently drive postural or muscular adaptations of the cervical spine over time, or alternatively, a pre-existing reduction in cervical lordosis could itself contribute to airway collapsibility and thus predispose to OSA. Furthermore, the observed association may be at least partly explained by unmeasured third variables that influence both cervical alignment and OSA risk independently, such as age-related degenerative changes, body composition/adiposity (as noted above regarding BMI), or general postural and musculoskeletal factors. The present cross-sectional data cannot distinguish between these possibilities, and longitudinal or interventional studies would be required to establish temporal precedence and causal direction.

The additional findings from this study show that both angular (C2–C7 Cobb angle, C7 slope) and translational (C2–C7 SVA) parameters of the subaxial cervical spine differed significantly between OSA patients and controls. C7 slope was significantly lower in the OSA group compared with controls (*p* = 0.015), while C2–C7 SVA was significantly higher (*p* = 0.016). However, neither C7 slope (ρ = −0.021, *p* = 0.911) nor C2–C7 SVA (ρ = +0.271, *p* = 0.148) showed a significant correlation with AHI within the OSA group. This pattern is consistent with that observed for the C2–C7 angle and suggests that these sagittal parameters may be associated with the presence of OSA rather than exhibiting a linear relationship with disease severity. While this observation is compatible with our proposed ‘threshold effect’ hypothesis, this interpretation should be regarded as preliminary given the limited sample size and cross-sectional design, and warrants confirmation in larger, longitudinal studies.

To eliminate the confounding effect that may arise from the lack of BMI data, additional soft tissue and airway measurements were performed on the same lateral cephalometric radiographs. Prevertebral soft-tissue thickness at the C2 level (PST-C2), used as an anatomical surrogate for local adiposity/obesity [31], was significantly higher in the OSA group compared to controls (4.93 ± 1.06 mm vs. 4.30 ± 0.70 mm, *p* = 0.017); this finding provides indirect anatomical evidence that OSA patients have greater regional soft tissue/fat accumulation, even in this cohort where BMI could not be directly measured. In contrast, no significant difference was found between groups for the same parameter measured at the C4 level (PST-C4) (6.31 ± 1.93 mm vs. 6.77 ± 2.00 mm, *p* = 0.321); this suggests that soft tissue/fat accumulation is not homogeneously distributed along the cervical spine, and may be more pronounced at upper cervical levels closer to the submental/submandibular region [32]. Importantly, neither PST-C2 (ρ = −0.164, *p* = 0.387) nor PST-C4 (ρ = −0.218, *p* = 0.247) showed a significant correlation with the C2–C7 angle. However, this absence of correlation should be interpreted cautiously: as PST-C2 is only an indirect and imperfect anatomical proxy for adiposity, a non-significant correlation between PST-C2 and cervical alignment does not, by itself, establish that BMI/adiposity does not confound the observed relationship. It merely indicates that regional soft-tissue thickness at the measured cervical levels- as opposed to systemic adiposity itself— is not a plausible imaging artifact underlying the angular findings. A true confounding effect of BMI on the cervical alignment–OSA relationship cannot be excluded without direct BMI data, and this remains an important limitation of the study.

To address the additional criticism regarding the lack of airway dimension assessment, the retroglossal pharyngeal airway space (PAS)— defined as the narrowest anteroposterior distance between the base of the tongue and the posterior pharyngeal wall at the level of the mandibular plane (Go-Me)— was re-measured in all OSA patients [19]. No significant difference was found between the OSA and control groups (8.63 ± 2.04 mm vs. 11.08 ± 5.43 mm, *p* = 0.154), and PAS also showed no significant correlation with AHI (Spearman ρ = −0.128, *p* = 0.500). This null finding should be interpreted with caution: given the modest sample size and the wide variability observed in the control group (SD = 5.43 mm), the study may simply have been underpowered to detect a true difference in this parameter, and the absence of a significant association does not by itself confirm that pharyngeal airway space is mechanistically uninvolved. Rather than being presented as evidence supporting a neuromuscular over a static anatomical mechanism, this finding is more appropriately regarded as inconclusive, and it should not be used to rule out anteroposterior airway narrowing as a contributing factor. Furthermore, it should be acknowledged as a limitation that, due to the two-dimensional nature of lateral cephalometric imaging, only the anteroposterior distance of the airway is reflected rather than its true cross-sectional area; therefore, three-dimensional cross-sectional area differences not detectable by this method may contribute additionally [6].

No significant difference was found between groups for the distance from the hyoid bone to the mandibular plane (MP-H) (20.40 ± 4.20 mm vs. 20.86 ± 5.30 mm, *p* = 0.832). The hyoid bone is anatomically connected to the mandible, tongue base, and- via the thyrohyoid membrane— to the larynx and pharyngeal wall through the suprahyoid and infrahyoid muscle chains; hyoid position is therefore used in the literature as an indirect indicator of the pharyngeal–laryngeal complex [23]. The absence of a significant difference in MP-H suggests that the change in cervical sagittal alignment does not markedly affect hyoid position, implying that the cervical lordosis–OSA relationship may operate through direct pharyngeal wall/muscle tone rather than through a hyoid-mediated mechanism [7]. However, this interpretation is a speculative hypothesis based on a single negative finding and requires confirmation through dynamic or functional imaging methods (e.g., real-time MRI during sleep); therefore, the proposed mechanism should be presented not as a definitive causal claim but as a hypothesis for future research.

The fact that our control group consisted of patients presenting with neck pain rather than asymptomatic individuals, albeit with radiologically normal cervical alignment, constitutes an important limitation. While this choice was made on ethical grounds to avoid unnecessary radiation exposure, it cannot be fully excluded that part of the observed between-group differences reflects the underlying symptomatic condition rather than OSA-specific pathology. Accordingly, our findings should be interpreted cautiously until confirmed against an asymptomatic reference population.

This study has several limitations. First, the cross-sectional design does not allow for causal inference. Second, the sample size is relatively small, particularly in the mild OSA subgroup (*n* = 6). Third, and most importantly, the control group was collected in a different time period (2023–2024) than the OSA patients (2017–2018); this temporal gap arose for two compounding reasons. First, at the time the original data collection was designed as part of a master’s thesis, the thesis committee did not mandate a concurrently recruited control group, and identifying an eligible, condition-matched control cohort meeting the same strict exclusion criteria proved practically difficult. Second, the subsequent COVID-19 pandemic further delayed prospective control recruitment, extending the gap until 2023–2024. Combined with the marked sex imbalance between groups (86.7% male in the OSA group vs. 43.3% male in the control group; *p* = 0.001), this should be regarded as a primary threat to internal validity rather than a secondary limitation, since a comparison this central ideally requires a contemporaneous, condition-matched control group. Although all radiographs in both periods were acquired using the same standardized cephalometric protocol, and our sensitivity analysis restricted to male participants together with the sex/age-adjusted ANCOVA models indicated that the observed differences remained significant independent of sex, neither approach can fully account for era-related effects. A prospective, contemporaneous, and sex-matched control cohort is therefore needed to confirm these findings. Fourth, and importantly, BMI data were not collected for either the OSA or control group in the original study design, representing a significant potential confounder given that obesity is a well-established risk factor for OSA. To partially address this limitation, prevertebral soft-tissue thickness (PST-C2, PST-C4) was measured on the same radiographs as an anatomical surrogate marker. While PST-C2 was significantly higher in the OSA group (*p* = 0.017), it did not correlate significantly with either AHI (ρ = 0.142, *p* = 0.453) or the C2–C7 angle (ρ = −0.164, *p* = 0.387) within the OSA group; this indicates that PST-C2 is, at best, a weak and indirect anatomical proxy for adiposity and cannot fully substitute for direct BMI measurement. Fifth, the C1–C2 angle was measured using the atlas tubercle line rather than an endplate-to-endplate reference, a method that is less standardized across the cephalometric literature than the C2–C7 Cobb angle; this may partly explain why the C1–C2 angle behaved differently from the other cervical parameters and showed no meaningful correlation with AHI. Sixth, although we were able to retrospectively measure C7 slope and C2–C7 SVA to provide a more comprehensive assessment of subaxial sagittal balance, T1 slope and full cranio-cervical alignment could not be reliably measured, as the field of view of the cephalometric radiographs used in this study did not consistently include the T1 vertebra in all subjects; this precluded a more complete characterization of global cervical sagittal balance. Consequently, the extent to which obesity may confound the observed cervical alignment–OSA relationship cannot be definitively excluded, and future studies should prospectively collect BMI data alongside these anatomical parameters.

## 5. Conclusions

This study demonstrated that cervical lordosis angles, particularly at the C2–C7 segment, were significantly reduced in patients with OSA compared to controls and showed a significant negative correlation with disease severity. Supplementary soft tissue and airway measurements suggest that this relationship is not simply reducible to differences in regional soft-tissue thickness or airway space narrowing at the measured cephalometric points, supporting a distinct, posture-related component of OSA pathophysiology; however, in the absence of direct BMI data, a confounding contribution of systemic adiposity to the observed cervical alignment–OSA association cannot be excluded. These findings warrant further investigation, ideally through longitudinal and functional imaging studies with larger, sex-matched cohorts, to clarify the mechanistic role of cervical posture in upper airway dynamics.

## Figures and Tables

**Table 1:** Demographic characteristics and cervical lordosis angles of study participants.

Characteristic	OSA Group (*n* = 30)	Control Group (*n* = 30)	Difference Estimate (95% CI)	*p*-Value	δ
Age, years	46 ± 11.3 44 [16.3]	40.7 ± 15.6 37.5 [13.8]	—	0.143	—
Sex, male, *n* (%)	26 (86.7)	13 (43.3)	—	0.001	—
C1–C2 °	25.51 ± 6.47 25.6 [9.36]	33.3 ± 4.44 33.0 [5.05]	− 7.44 (−10.64 to −4.26)	<0.001	−0.66
C1–C7 °	35.0 ± 11.9 34.0 [14.3]	51.2 ± 7.34 52.0 [7.63]	−16.27 (−21.15 to −11.62)	<0.001	−0.80
C2–C7 °	9.19 ± 10.6 8.56 [12.9]	19.9 ± 5.05 19.5 [5.77]	−10.80 (−14.45 to −6.98)	<0.001	−0.69
C7 slope (°)	20.90 [18.70–29.48]	26.95 [24.90–31.05]		0.015	−0.37
C2–C7 SVA (mm)	26.86 [23.43–34.48]	22.05 [16.57–27.27]		0.016	+0.36

Data are presented as mean ± standard deviation and median [interquartile range] for continuous variables, or as counts and percentages for categorical variables. Difference estimates with 95% confidence intervals are reported for cervical angle comparisons. Cliff’s delta (δ) was used as the nonparametric effect size for Mann–Whitney U comparisons; negative values indicate lower cervical lordosis angles in the OSA group relative to controls.

**Table 2:** Cervical lordosis angles according to OSA severity subgroup.

Angle	Mild OSA (*n* = 6)	Moderate OSA (*n* = 11)	Severe OSA (*n* = 13)	*p*-Value	ε^2^
C1–C2, °	23.5 ± 7.28 25.9 [10.3]	26.3 ± 5.31 26.4 [8.76]	25.8 ± 7.28 25.0 [11.3]	0.739	0.02
C1–C7, °	41.3 ± 10.8 42.4 [3.94]	34.5 ± 8.41 36.5 [9.25]	32.5 ± 14.5 29.7 [8.58]	0.133	0.14
C2–C7, °	17.2 ± 4.85 19.2 [4.23]	8.02 ± 8.17 10.4 [9.49]	6.49 ± 12.8 5.17 [6.24]	0.019 ^a,b^	0.28

Data are presented as mean ± standard deviation and median [interquartile range]. ε^2^ = epsilon-squared effect size. ^a^: significant difference between mild and moderate OSA (*p* < 0.05); ^b^: significant difference between mild and severe OSA (*p* < 0.05).

**Table 3:** Intraclass correlation coefficients (ICC) for cervical lordosis angle measurements.

Cervical Angle	Group	ICC (2,1)	95% CI	Interpretation
C1–C2	OSA (*n* = 15)	0.972	0.921–0.991	Excellent
C1–C2	Control (*n* = 15)	0.934	0.831–0.979	Excellent
C1–C7	OSA (*n* = 15)	0.932	0.811–0.977	Excellent
C1–C7	Control (*n* = 15)	0.979	0.935–0.992	Excellent
C2–C7	OSA (*n* = 15)	0.922	0.772–0.971	Excellent
C2–C7	Control (*n* = 15)	0.968	0.963–0.996	Excellent

ICC = Intraclass correlation coefficient; two-way random effects model, single measures, absolute agreement [ICC(2,1)]. ICC values were calculated on a randomly selected subsample of 15 participants per group. Reliability was interpreted as: poor (<0.50), moderate (0.50–0.75), good (0.75–0.90), and excellent (>0.90).

**Table 4:** Spearman rank correlation coefficients between AHI and cervical lordosis angles within the OSA Group.

Cervical Angle	Spearman ρ	*p*-Value	Interpretation
C1–C2, °	+0.118	0.534	No significant correlation
C1–C7, °	−0.361	0.050	Borderline, not significant
C2–C7, °	−0.522	0.003	Moderate negative
C7 slope, °	−0.021	0.911	No significant correlation (very weak)
C2–C7 SVA, mm	+0.271	0.148	Weak, not significant

Correlations were calculated within the OSA group only (*n* = 30) using continuous AHI values. AHI = Apnea–Hypopnea Index; ρ = Spearman’s rank correlation coefficient.

**Table 5:** Sensitivity analysis: between-group differences in cervical lordosis angles restricted to male participants (Mann–Whitney U Test).

Angle	OSA Males	Control Males	*p*-Value
C1–C2	25.68 ± 6.60°	32.00 ± 3.56°	0.006
C1–C7	35.18 ± 12.81°	49.45 ± 6.37°	<0.001
C2–C7	9.61 ± 11.11°	20.18 ± 5.86°	0.002

**Table 6:** Comparison of soft tissue and airway parameters between OSA and control groups.

Parameter	OSA Group (*n* = 30) Mean ± SD	Control Group (*n* = 29) Mean ± SD	*p*-Value	Cliff’s δ
PST-C2 (mm)	4.93 ± 1.06	4.30 ± 0.70	**0.017**	0.363
PST-C4 (mm)	6.31 ± 1.93	6.77 ± 2.00	0.321	−0.152
PAS (mm)	8.63 ± 2.04	11.08 ± 5.43	0.154	−0.217
MP-H (mm)	20.40 ± 4.20	20.86 ± 5.30	0.832	−0.033

Mann–Whitney U test; Cliff’s δ effect size reported given non-normal distribution. Bold indicates statistical significance (*p* < 0.05). (SD: standard deviation).

**Table 7:** Correlation of soft tissue/airway parameters with OSA severity (AHI) and C2–C7 angle (within OSA group, *n* = 30).

Parameter	ρ vs. AHI	*p*-Value	ρ vs. C2–C7 Angle	*p*-Value
PST-C2	0.142	0.453	−0.164	0.387
PST-C4	0.084	0.658	−0.218	0.247
PAS	−0.128	0.500	−0.095	0.619
MP-H	−0.001	0.995	0.163	0.389

Spearman’s rho reported. No significant correlations were observed for any parameter.

**Table 8:** Multiple linear regression (ANCOVA) models predicting cervical lordosis angles, adjusted for age and sex (*n* = 60).

Outcome	Predictor	β	95% CI	*p*-Value
C1–C2	Group (OSA vs. Control)	−6.98	−10.29 to −3.67	<0.001
	Age	−0.05	−0.16 to 0.06	0.343
	Sex (M vs. F)	−1.31	−4.72 to 2.10	0.444
C1–C7	Group (OSA vs. Control)	−15.92	−21.88 to −9.96	<0.001
	Age	0.05	−0.14 to 0.25	0.584
	Sex (M vs. F)	−1.49	−7.62 to 4.65	0.629
C2–C7	Group (OSA vs. Control)	−11.79	−16.74 to −6.83	<0.001
	Age	0.08	−0.09 to 0.24	0.351
	Sex (M vs. F)	1.53	−3.57 to 6.63	0.549
C7 slope	Group (OSA vs. Control)	−3.97	−8.10 to 0.17	0.060
	Age	0.04	−0.10 to 0.17	0.575
	Sex (M vs. F)	0.52	−3.74 to 4.78	0.808
C2–C7 SVA	Group (OSA vs. Control)	+5.52	0.23 to 10.80	0.041
	Age	−0.02	−0.19 to 0.16	0.850
	Sex (M vs. F)	1.87	−3.57 to 7.30	0.494

Linear regression, *n* = 60 (30 OSA, 30 control); reference categories: control group, female sex.

**Table 9:** Between-group comparison of C7 slope and C2–C7 sagittal vertical axis (SVA).

Parameter	OSA Group (*n* = 30) Mean ± SD; Median [IQR]	Control Group (*n* = 30) Mean ± SD; Median [IQR]	*p*-Value (Mann–Whitney U)	Cliff’s δ
C7 slope, °	24.14 ± 8.79; 20.9 [10.78]	27.68 ± 4.12; 26.95 [6.15]	0.015	−0.37
C2–C7 SVA, mm	28.50 ± 9.54; 26.86 [11.05]	22.26 ± 7.95; 22.05 [10.70]	0.016	+0.36
Parameter	OSA Group (*n* = 30) Mean ± SD; Median [IQR]	Control Group (*n* = 30) Mean ± SD; Median [IQR]	*p*-value (Mann–Whitney U)	Cliff’s δ

Mann–Whitney U test; Cliff’s δ effect size reported given non-normal distribution. Negative δ indicates lower values in OSA group; positive δ indicates higher values in OSA group.

## Data Availability

The datasets generated and analyzed during the current study are available from the corresponding author upon reasonable request, subject to ethical approval and data protection regulations.
